# Is body mass index a risk factor for low cerebral oxygenation during spinal anesthesia in women undergoing cesarean section? A preliminary study

**DOI:** 10.3906/sag-1810-208

**Published:** 2019-06-18

**Authors:** Gülçin AYDIN, Cemile Dayangan SAYAN

**Affiliations:** 1 Department of Anesthesiology and Reanimation, School of Medicine, Kırıkkale University, Kırıkkale Turkey; 2 Department of Obstetrics and Gynecology, School of Medicine, Kırıkkale University, Kırıkkale Turkey

**Keywords:** Body mass index, cerebral oxygenation, near-infrared spectroscopy, cesarean section

## Abstract

**Background/aim:**

In this study, it was aimed to investigate the relationship between body mass index (BMI) and cerebral oxygenation during spinal anesthesia in women undergoing cesarean section. This study also aimed to demonstrate whether fetal cord blood oxygenation was affected by maternal BMI and/or delivery time.

**Materials and methods:**

The study included parturients with ASA I score undergoing cesarean section under spinal anesthesia in 2015 and 2016. They were divided into two groups according to BMI: Group 1 comprised parturients with BMI of <30 (n = 11) and Group 2 comprised parturients with BMI of ≥30 (n = 17). Right cerebral oxygenation (RSO2) and left cerebral oxygenation (LSO2) monitoring was performed using near-infrared spectroscopy (NIRS). The participants were divided into two groups according to the duration of fetal delivery. Group A included parturients with delivery time of <2 min (n = 7) and Group B those with delivery time of >2 min (n = 18), and fetal cord blood oxygenation was measured using a blood gas analyzer.

**Results:**

Evaluation was made of a total of 25 patients. The RSO2 values were​​ measured at the 20th, 30th, and 35th minutes of the cesarean section procedure and the median values of all the time intervals in Group 1 were significantly lower than those of Group 2 (P < 0.05). The LSO2 value ​​was significantly lower in Group 1 at the 35th minute compared to Group 2 (P < 0.05). The PO2 values of fetal cord blood were significantly lower in Group B (P < 0.05).

**Conclusion:**

The results of this study showed that parturients with BMI of <30 who are undergoing cesarean section under spinal anesthesia might have an increased risk of complications due to decreased cerebral oxygenation related with hypotension. Therefore, it can be suggested that before and during cesarean section these patients should be closely monitored for cerebral oxygenation using NIRS.

## 1. Introduction

Hypotension is a relatively frequent complication of spinal anesthesia in women undergoing cesarean section with incidence ranging from 50% to 80%. Hypotension during cesarean section is believed to be due to the blockade of regional sympathetic activity [1]. Parturients with hypotension may present with nausea, vomiting, and unconsciousness, and if it persists, pulmonary aspiration may occur. The fetus may also be affected, and hypoxia and acidosis may be detected in cord blood [2,3]. Therefore, to avoid hypotension, various strategies have been attempted, including intravenous fluid administration, prophylactic application of vasoactive drugs, and positioning of the patient [4,5]. Near-infrared spectroscopy (NIRS) is being increasingly used for noninvasive monitoring of cerebral oxygenation in many clinical scenarios [6]. During elective cesarean delivery under spinal anesthesia, NIRS has demonstrated >5% decrease in cerebral oxygen saturation from baseline [7]. Furthermore, regional cerebral blood oxygenation and regional cerebral blood volume have also been shown to decrease by 4% and 6 µmol/L, respectively, from baseline [8].

It has been reported in the literature that maternal under- or overweight status is known to alter placental development and it is agreed that fetal hypoxemia due to undernutrition could be seen in underweight mothers [9]. Moreover, antenatal anemia, preterm delivery, and low birth weight have been reported to be seen more frequently in women with a low body mass index (BMI) than in women with a normal BMI [10]. However, to the best of our knowledge, there are no data in the literature describing the relationship between maternal BMI and maternal cerebral oxygenation during spinal anesthesia administered for cesarean section. 

In light of the above findings, the aim of this study was to investigate the relationship between BMI and cerebral oxygenation during spinal anesthesia in women undergoing cesarean section. It was also aimed to demonstrate whether fetal cord blood oxygenation was affected by maternal BMI and/or delivery time.

## 2. Materials and methods 

### 2.1. Participants

The study was conducted between August 2015 and August 2016 after receiving approval from the local ethics committee (No: 12/04, 11.05.2015). All participants provided an informed consent form. 

The study included pregnant women aged 18–50 years with an American Society of Anesthesiologists (ASA) score of I, who were undergoing elective cesarean section under spinal anesthesia. Age and BMI values on admission were recorded for all participants before performing the spinal anesthesia. Patients with ASA scores of II–IV and patients who had sepsis and bacteremia, skin infection at the injection site, severe hypovolemia, coagulopathy, treatment with anticoagulant therapy, and/or increased intracranial pressure were excluded from the study. In addition, patients who did not accept spinal anesthesia were excluded.

On first admission, a record was made for each participant of age (years), BMI (kg/m2), height (cm), blood hemoglobin (/µL), and hematocrit (%) levels before the administration of spinal anesthesia. Since the participants were selected randomly and prenatal maternal follow-up information could not be obtained from some patients, prenatal maternal follow-up information could not be recorded and analyzed in this study. As the BMI levels of all the participants in the study ranged between 23.60 and 42.97 and the median BMI value was calculated as 31.20, the cut-off value for BMI was defined as 30 and the participants were divided into two groups according to BMI values as follows:

- Group 1 (BMI <30, n = 11), 

- Group 2 (BMI ≥30, n = 14).

The participants were also divided into two groups according to the duration of fetal delivery, as follows: 

- Group A comprised parturients with delivery time of <2 min (n = 7), 

- Group B comprised parturients with delivery time of >2 min (n = 18). 

To determine if fetal oxygenation was affected by maternal BMI and/or delivery time, fetal cord blood oxygenation was measured using a blood gas analyzer (Siemens RAPIDlab 1265, USA).

### 2.2. Spinal anesthesia procedure 

Before administering the spinal anesthesia, the heart rate (HR), noninvasive blood pressure (NIBP), and peripheral oxygen saturation (SpO2) were monitored while the patients were in the lateral tilt position. Then 8 mL/kg intravenous fluid (Ringer’s lactate) was administered to patients within 20 min via an 18- or 20-G catheter. Spinal anesthesia was applied while the patients were in the sitting position. A 25-G Quincke spinal needle (Spinocan, B. Braun, USA) was inserted in the L4–5 intervertebral space. Once free flow of clear cerebrospinal fluid was observed, 0.5% hyperbaric bupivacaine (Marcaine, spinal heavy, 0.5%, Astra Zeneca, Turkey) was injected in 10 s. Then the patient was positioned supine. The motor block level was tested using the modified Bromage scale (0 = no paralysis, 1 = inability to raise extended leg, 2 = inability to flex knee, 3 = inability to dorsiflex foot but can wiggle toes, 4 = inability to move at all), and the sensorial block was tested with the pin-prick test [u290e]. The operation was started when the sensorial block reached the T4 level. All the participants were monitored for blood pressure (systolic blood pressure [SBP], diastolic blood pressure [DBP], and mean blood pressure [MBP]), HR, and SpO2 at baseline, within the first minute, and at every 5 min during anesthesia. When oxygen saturation of the patient fell below 90% during the delivery period, it was decided to apply oxygen support to the patient with a nasal mask at a rate of 4 L/min. Patient intubation was predicted if the patient’s oxygen saturation continued to decline. Hypotension was defined as SBP of <90 mmHg or a decrease of ≥20% from baseline and was treated with 5–10 mg of intravenous ephedrine and rapid fluid replacement as required. 

### 2.3. Cesarean section procedure

All surgical procedures were carried out by two obstetricians. Povidone iodine was used for surgical cleansing of the skin and 1 g of IV cefazolin (Sefazol, u2911, İstanbul, Turkey) was administered to all patients as prophylaxis during the skin incision. The cesarean section was performed with a Pfannenstiel abdominal incision and a transverse incision of the lower uterine segment. A slight and sharp dissection of the vesico-uterine peritoneal fold was performed. Closure of the low transverse uterine incision was made using a single-layer locked suture, including the decidua, with the visceral peritoneum open and the parietal peritoneum closed.

### 2.4. Umbilical cord blood analysis

The umbilical cord blood of the newborn was sampled with a heparinized syringe and analyzed just after birth using a blood gas analyzer as described above. 

### 2.5. Cerebral oxygenation monitoring 

Cerebral oxygenation (SO2) monitoring was performed using a NIRS device (INVOS Somanetics 5100, Troy, MI, USA) by placing the probes over the right (RSO2) and left (LSO2) frontal regions, 1 cm above the eyebrow and 1 cm lateral to the midline and distant from the temporalis muscle. Normal RSO2 and LSO2 values are 60% or higher in the brain tissue [12]. The hemodynamic measurements and NIRS data were recorded at baseline and every 5 min during surgery. 

### 2.6. Statistical analysis

Statistical analysis was performed using SPSS 21.0 Windows (IBM Corp., Armonk, NY, USA). As the variables did not show normal distribution in the groups, the Mann–Whitney U test was used for the comparison of parameters between groups. Correlation analysis was performed using Spearman’s rho correlation test. P < 0.05 was accepted as statistically significant. 

Power analysis of this study was applied using the Gpower 3.1 package program. Following the measurements using mean and standard deviation data of the BMI parameters of the groups, the following results were obtained: effect size d = 2.07; power = 0.95; minimum total number of participants to be included in this study = 12.

## 3. Results

The clinical and demographic features are summarized in Table 1. The study included a total of 25 patients with a mean age of 28.52 ± 5.31 years (range: 19–39 years). None of the patients had nausea, vomiting, or unconsciousness during spinal anesthesia. 

**Table 1:** The clinical and demographic features of the groups. Mann–Whitney U test, P < 0.05.

Variable	Group 1 (n = 11)	Group 2 (n = 14)	P
Age (years)	26.45 (23.0–33.0)	30.14 (19.0–39.0)	0.099
Height (cm)	167.36 (160.0–173.0)	163.92 (151.0–173.0)	0.099
Weight (kg)	76.90 (68.0–85.0)	91.50 (79.0–110.0)	<0.001
BMI	27.44 (23.60–29.74)	34.15 (30.41–42.97)	<0.001
Hemoglobin (/µL)	11.80 (10.60–13.20)	12.10 (10.70–14.10)	0.546
Hematocrit (%)	35.03 (29.0–39.0)	35.78 (29.0–40.0)	0.392
Fetal delivery time (min)	2.54 (1.0–5.0)	2.50 (1.00–5.00)	0.933
Duration of surgery (min)	31.45 (20.0–40.0)	34.07 (25.0–40.0)	0.416
Fetal weight (g)	3000.90 (570.0–3955.0)	3324.61 (2700.0–4410.0)	0.434
Fetal hemoglobin (/µL)	16.13 (13.30–18.0)	15.83 (12.50–17.10)	0.601
Fetal pH	7.35 (7.27–7.43)	7.35 (7.18–7.45)	0.861
Fetal PO2	21.10 (9.40–32.70)	25.00 (7.30–33.10)	0.885

n: Number of participants, BMI: body mass index, PO2: O2 partial pressure. Bold P-values denote significance. Data are given as median (25%–75%).

The BMI and weight values were significantly higher in Group 2 compared to Group 1 (P < 0.05). No significant difference was observed between the groups in terms of age, height, hemoglobin (Hb), hematocrit (Hct), duration of surgery, duration of delivery, fetal weight, fetal Hb, fetal pH, or fetal PO2. 

**Figure 1:**
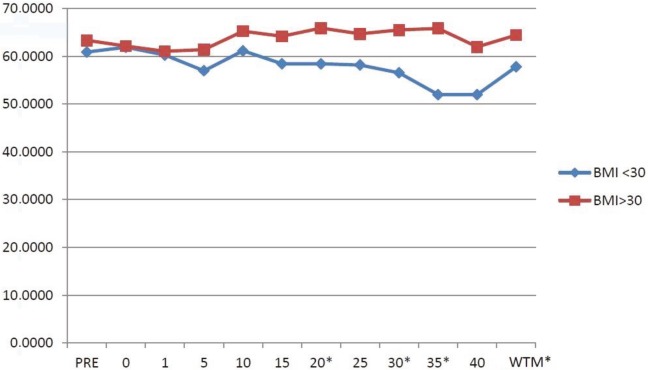
Brain right hemisphere oxygenation (RSO2) of the groups. Data are given as median (25%–75%). *P < 0.05, WTM = whole time median.

The RSO2 and LSO2 values of the groups are shown in Table 2. In Group 1, the RSO2 values measured at the 5th, 15th, 20th, 25th, 30th, 35th, and 40th minutes were <60%. At all times, the RSO2 values in Group 2 were measured as >60%. The RSO2 values measured at the 20th, 30th, and 35th minutes and the median values of all the time intervals​​ in Group 1 were significantly lower than those of Group 2 (P < 0.05) (Figure 1). 

**Table 2:** The values of the near-infrared spectroscopy (NIRS), peripheral oxygen saturation, and arterial pressure measurements of the groups. Mann–Whitney U test, P < 0.05.

Variables	Group 1	Group 2	P
RSO2, 20th minute	58.45 (49.00–68.00)	65.92 (59.00–95.00)	0.032
RSO2, 30th minute	56.57 (46.00–70.00)	65.53 (55.00–75.00)	0.019
RSO2, 35th minute	52.00 (42.00–61.00)	65.88 (52.00–78.00)	0.017
RSO2, whole time median	57.82 (48.71–66.00)	64.46 (47.89–73.90)	0.025
LSO2, 35th minute	55.00 (48.00–65.00)	62.87 (57.00–71.00)	0.017
LSO2, whole time median	57.82 (47.57–69.29)	62.82 (47.67–70.71)	0.043
Saturation, 10th minute	99.18 (98.00–100.00)	98.21 (96.00–100.00)	0.032
Saturation, 35th minute	99.83 (99.00–100.00)	98.12 (95.00–100.00)	0.011
SBP, 20th minute	92.90 (77.00–121.00)	105.64 (80.00–135.00)	0.021
DBP, 1st minute	50.81 (32.00–73.00)	64.21 (42.00–84.00)	0.020
DBP, 5th minute	48.36 (32.00–57.00)	61.00 (40.00–85.00)	0.003
MBP, 5th minute	65.36 (49.00–91.00)	77.92 (54.00–108.00)	0.025
MBP, 15th minute	66.81 (43.00–90.00)	78.28 (54.00–94.00)	0.014

RSO2: Oxygen saturation of the right cerebral hemisphere, LSO2: oxygen saturation of the left cerebral hemisphere, SBP: systolic blood pressure, DBP: diastolic blood pressure, MBP: mean blood pressure. Bold P-values denote significance. Data are given as median (25%–75%).

**Figure 2:**
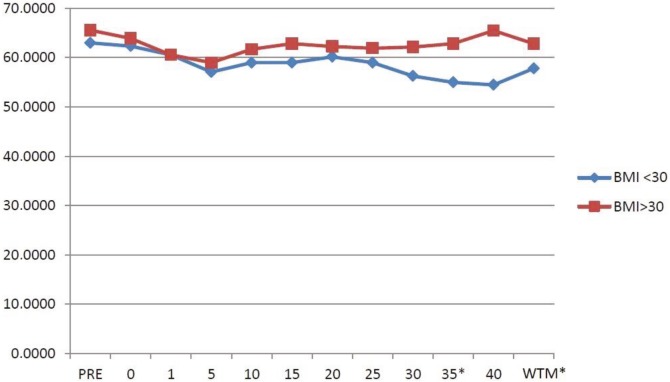
Brain left hemisphere oxygenation (LSO2) of the groups. Data are given as median (25%–75%). *P < 0.05, WTM = whole time median.

In Group 1, the LSO2 values measured at the 5th, 10th, 15th, 25th, 30th, 35th, and 40th minutes were <60%. In Group 2, the LSO2 values​​ measured at all the time intervals with the exception of the 5th minute were >60%. In Group 1, the LSO2 value ​​measured at the 35th minute and the median values of all the time intervals were significantly lower than those of Group 2 (P < 0.05) (Figure 2).

The correlation analysis results demonstrated a positive correlation between the BMI and SBP values measured at the 15th and 20th minutes, between the BMI and DBP values measured at the 15th minute, and between the BMI and MBP values measured at the 15th minute (P < 0.05) (Table 3). A positive correlation was found between the maternal weight and RSO2 values, between the maternal weight and LSO2 values, between the BMI and RSO2 values, and between BMI and LSO2 values****(Table 4).

**Table 3:** The relationships between the maternal body mass index (BMI) and hemodynamic parameters. Spearman’s rho correlation test, P < 0.05.

Hemodynamic parameters		BMI
SBP, 15th minute	r	0.426
	P	0.034
SBP, 20th minute	r	0.495
	P	0.012
DBP, 15th minute	r	0.404
	P	0.045
MBP, 15th minute	r	0.544
	P	0.005

BMI: Body mass index, SBP: systolic blood pressure, DBP: diastolic blood pressure, MBP: mean blood pressure.

**Table 4:** This table demonstrates a positive correlation between NIRS and patient weight, and between NIRS and maternal BMI. Spearman’s rho correlation test, P < 0.05.

Variable		Weight	BMI
RSO2, 15th minute	r	0.503	0.448
	P	0.010	0.025
RSO2, 20th minute	r	0.479	0.443
	P	0.015	0.027
RSO2, 25th minute	r	0.516	0.521
	P	0.010	0.009
RSO2, 30th minute	r	0.616	0.576
	P	0.004	0.008
RSO2, 35th minute	r	0.845	0.828
	P	<0.001	<0.001
RSO2, 40th minute	r	0.868	0.638
	P	0.025	0.173
RSO2, whole time mean	r	0.426	0.479
	P	0.034	0.015
LSO2, 35th minute	r	0.771	0.808
	P	0.001	<0.001
LSO2, 40th minute	r	0.464	0.903
	P	0.354	0.014
LSO2, whole time mean	r	0.426	0.411
	P	0.034	0.041

BMI: Body mass index, RSO2: oxygen saturation of the right cerebral hemisphere, LSO2: oxygen saturation of the left cerebral hemisphere. Bold P-values denote significance.

When the patients were divided into two groups according to the duration of fetal delivery as <2 min (Group A) and >2 min (Group B), the partial oxygen saturation (PO2) of the cord blood was significantly lower in Group B (P < 0.05) (Table 5). No correlation was found between maternal BMI and fetal cord blood oxygenation. No correlation was found between the maternal NIRS values and fetal cord blood oxygenation levels.

**Table 5:** The values of the blood gas parameters according to the fetal delivery time. Mann–Whitney U test, P < 0.05.

Variable	Group A (n = 7)	Group B (n = 17)	P
pH	7.35 (7.23–7.41)	7.33 (7.18–7.45)	0.425
PO2	29.31 (25.00–33.10)	18.18 (7.30–30.20)	0.003
PCO2	36.74 (31.00–48.30)	42.08 (31.10–74.00)	0.280
HCO3	21.47 (19.50–25.00)	22.95 (16.90–29.00)	0.340
BE	–3.90 (–7.80 to 1.90)	–2.07 (–5.60 to 4.60)	0.162

PO2: O2 partial pressure, PCO2: CO2 partial pressure, HCO3: bicarbonate level, BE: base excess value, n: number of fetuses. Bold P-values denote significance.

## 4. Discussion

NIRS is an easy-to-use tool providing noninvasive measurement of cerebral oxygenation. However, there have been some concerns with respect to its clinical value as the technique has high intra- and interindividual variability as seen in a study by Thavasthy et al. [13], which reported mean 62.3 ± 6.0% baseline values for RSO2 in healthy individuals. Cerebral oxygenation measurement using NIRS has previously been investigated in both parturients undergoing spinal anesthesia for cesarean section and neonates [1–5,7,8,13]. The results of the current study demonstrated that RSO2 and LSO2 were >60% in parturients with BMI of >30 and generally <60% in those with BMI of <30. According to these results, it could be said that cerebral oxygenation was low in patients with BMI of <30 compared to those with BMI of >30. Furthermore, SBP at the 20th minute, DBP at the 1st and 5th minutes, and MBP at the 5th and 15th minutes were lower in patients with BMI of <30. Significant correlations were determined between maternal weight and RSO2 and LSO2 values and between the maternal BMI and RSO2 and LSO2 values. The values of the SBP, DBP, MBP, RSO2, and LSO2 were measured to be low in parturients with BMI of <30. However, no significant difference was observed between the groups in terms of cerebral oxygenation during the period in which hemodynamic differences were present. There was found to be a positive correlation between BMI and some of the hemodynamic parameters; therefore, a direct effect of BMI on cerebral oxygenation could not be confirmed. However, the current study revealed that women with BMI of <30 might have an increased risk of complications due to decreased cerebral oxygen saturation even if they are classified as ASA I. In light of these results, it can be suggested that pregnant women with BMI of <30 should be monitored for oxygen saturation using NIRS even if they are asymptomatic. Although the study groups consisted of parturients with ASA I score and no comorbidities, this decreased cerebral oxygenation in parturients with BMI of <30 may be associated with the following possible theoretical mechanisms: 1) low blood volume and blood pressure that may develop secondary to BMI of <30; 2) low blood volume and blood pressure secondary to pregnancy, anemia, and vitamin deficiencies that may occur due to malnutrition; 3) low blood volume and blood pressure due to anemia and vitamin deficiencies secondary to pregnancy. However, this situation could not be identified in this study and therefore it requires further investigations in future studies.

Unfortunately, cerebral oxygenation of the newborns was not measured in this study. However, a significant difference was determined between the groups with respect to cord blood PO2 when the groups were categorized according to the duration of fetal delivery (<2 min and >2 min). This issue was related to the prolonged delivery time rather than maternal BMI, because there was no relationship between maternal BMI and fetal cord blood oxygenation. Furthermore, there was no correlation between the maternal NIRS values and fetal cord blood oxygenation. From these results, it was thought that fetal cord blood oxygenation was protected even though maternal blood oxygenation was adversely affected during the delivery process.

Cerebral oxygenation is affected by several variables including cerebral blood volume, venous and capillary saturation, and the partition ratio between the arterial and venous components of the cerebral vasculature [14,15]. The association between the decrease in cerebral oxygen saturation after spinal anesthesia and hypotension was investigated using NIRS in a study by Hirose et al. [16]. A significant decrease was determined in mean oxy-Hb, total-Hb, tissue oxygenation index, and mean arterial pressure after spinal anesthesia with bupivacaine. It was suggested that the decrease in cerebral blood volume and cerebral blood oxygenation was associated with the severity of hypotension [16]. Similarly, in the current study, the MBP, RSO2, and LSO2 decreased after spinal anesthesia. However, unlike the study by Hirose et al. [16], the current study classified parturients according to BMI, and the SBP, DBP, MBP, RSO2, and LSO2 values were determined to be significantly lower in the group with BMI of <30 at most time points during spinal anesthesia. 

In a recent study, Sun et al. [17] investigated the role of cerebral oxygen saturation in the prediction of hypotension after spinal anesthesia. Of the 45 parturients included in the study, 32 had hypotension after spinal anesthesia. The decrease in cerebral oxygen saturation after spinal anesthesia in parturients with hypotension was greater than in those without hypotension (P < 0.05). It was suggested that the decrease in cerebral oxygen saturation might be a clinically useful predictor of hypotension after spinal anesthesia for cesarean section. In the current study, the decrease in SBP, DBP, MBP, and RSO2, and LSO2 values was more prominent in the parturients with BMI of <30. Although all the patients underwent the same spinal anesthesia procedure, parturients with BMI of <30 were prone to hemodynamic instability, while those with BMI of >30 were resistant to hypotension induced by spinal anesthesia. 

A major limitation of the current study is the relatively low number of included subjects. In addition, prenatal maternal follow-up information could not be recorded and analyzed in this study, since the participants were selected randomly and prenatal maternal follow-up information could not be obtained from some patients. Therefore, this study could not describe how the prenatal maternal weight gain affected the maternal and/or fetal cerebral oxygenation. Furthermore, although measurements were taken of the maternal cerebral blood oxygenation, if measurements had been taken of the newborns, further knowledge could have been provided about the effects of BMI in pregnancy on both maternal and fetal oxygenation. 

In conclusion, the results of this study demonstrated that parturients with BMI of <30 who are undergoing cesarean section under spinal anesthesia might have an increased risk of complications due to decreased cerebral oxygenation related to hypotension. Therefore, it can be suggested that these patients should be closely monitored for cerebral oxygenation using NIRS before and during cesarean section.

## Acknowledgments

The preliminary results of this study were presented at the 12th Congress of the Turkish-German Gynecological Society, Kyrenia, Turkish Republic of Northern Cyprus, in 2018. The study was financially supported by the Kırıkkale University Scientific Research Projects Coordination Unit (Project Number: 2015-057).
